# Artificial intelligence and machine learning techniques for suicide prediction: Integrating dietary patterns and environmental contaminants

**DOI:** 10.1016/j.heliyon.2024.e40925

**Published:** 2024-12-04

**Authors:** Mayyas Al-Remawi, Ahmed S.A. Ali Agha, Faisal Al-Akayleh, Faisal Aburub, Rami A. Abdel-Rahem

**Affiliations:** a Department of Pharmaceutics and Pharmaceutical Technology, Faculty of Pharmacy and Medical Sciences, University of Petra, Amman, Jordan; b School of Pharmacy, Department of Pharmaceutical Sciences, The University of Jordan, Amman, 11942, Jordan; c Faculty of Administrative & Financial Sciences University of Petra Amman, Jordan; d Faculty of Arts and Sciences, Department of Chemistry. University of Petra, Amman, Jordan

**Keywords:** Artificial intelligence (AI), Machine learning (ML), Suicide prediction, Environmental contaminants, Dietary influences

## Abstract

**Background:**

Suicide remains a leading cause of death globally, with nearly 800,000 deaths annually, particularly among young adults in regions like Europe, Australia, and the Middle East, highlighting the urgent need for innovative intervention strategies beyond conventional methods.

**Objectives:**

This review aims to explore the transformative role of artificial intelligence (AI) and machine learning (ML) in enhancing suicide risk prediction and developing effective prevention strategies, examining how these technologies integrate complex risk factors, including psychiatric, socio-economic, dietary, and environmental influences.

**Methods:**

A comprehensive review of literature from databases such as PubMed and Web of Science was conducted, focusing on studies that utilize AI and ML technologies. The review assessed the efficacy of various models, including Random Forest, neural networks, and others, in analyzing data from electronic health records, social media, and digital behaviors. Additionally, it evaluated a broad spectrum of dietary factors and their influence on suicidal behaviors, as well as the impact of environmental contaminants like lithium, arsenic, fluoride, mercury, and organophosphorus pesticides.

**Conclusions:**

AI and ML are revolutionizing suicide prevention strategies, with models achieving nearly 90 % predictive accuracy by integrating diverse data sources. Our findings highlight the need for geographically and demographically tailored public health interventions and comprehensive AI models that address the multifactorial nature of suicide risk. However, the deployment of these technologies must address critical ethical and privacy concerns, ensuring compliance with regulations and the development of transparent, ethically guided AI systems. AI-driven tools, such as virtual therapists and chatbots, are essential for immediate support, particularly in underserved regions.

## Highlights

• AI/ML integrate diverse data sources to refine suicide risk predictions.
• Dietary patterns have significant correlations with suicide risk factors.
• Certain water contaminants may contribute to reduced suicide incidence.
• AI-driven interventions provide real-time support in suicide prevention.
• ML personalizes suicide prevention strategies based on individual risk factors.

## 1 Introduction

Suicide remains a significant public health issue globally, with nearly 800,000 deaths annually [1,2]. This makes it the second leading cause of death among 15-29-year-olds worldwide [3]. In recent years, various regions have reported concerning trends. For instance, in Europe, the European Union reports approximately 56,000 suicides each year, with Lithuania and Slovenia experiencing some of the highest rates [4]. In Australia, suicide is the leading cause of death for individuals aged 15–44 [5], with over 3000 deaths annually [6]. Similarly, countries in the Middle East have witnessed a significant rise in suicide rates in suicide attempts reported over the past decade [7]. Despite cultural and regional differences, the rising trend is a common, highlighting a global crisis that requires immediate and effective intervention strategies.

Suicide is a multifactorial phenomenon with numerous potential causes. These can range from psychiatric conditions, such as depression and anxiety, to socio-economic factors, such as unemployment and financial distress [8,9]. Additionally, dietary habits and environmental factors like water and food contamination also play a significant role in influencing suicidal tendencies. For example, the combined use of alcohol and energy drinks has been shown to significantly increase the likelihood of suicidal ideation and attempts [10]. Similarly, certain contaminants in drinking water, such as lithium, have been associated with lower suicide rates, suggesting a protective effect [11].

Timely identification of suicide risk through predictive models (suicide prediction) and the subsequent implementation of intervention strategies (suicide prevention) are both crucial yet distinct steps in reducing suicide rates. Suicide prediction focuses on identifying individuals at high risk of attempting suicide based on various data inputs [12], while suicide prevention involves actions taken to mitigate this risk and provide appropriate care [13]. Conventional approaches, which heavily depend on clinical assessments and patient self-reports, frequently fail to identify individuals at risk [14]. Research suggests that these methods have a predictive accuracy of around 20–30 % [15]. Conventional methods may have varying perspectives, lack uniformity, and be influenced by the accessibility and reliability of mental health resources. It is worth noting that in the United States, a significant proportion of individuals who die by suicide do not have a documented mental health condition at the time of their death [16]. This highlights the shortcomings of conventional assessment methods.

Advances in artificial intelligence (AI) and machine learning (ML) offer promising new avenues for suicide prediction and prevention. AI technologies can analyze vast amounts of data from various sources—such as electronic health records (EHRs), social media, and wearable devices—to identify patterns and risk factors associated with suicidal behavior [17]. However, while some AI models have achieved up to 90 % predictive accuracies, it is essential to note that these high accuracies are typically achieved under specific conditions and are not universally applicable across all datasets and populations [18]. For example, AI models trained on structured clinical data, such as EHRs, often perform better than those trained on unstructured social media data, which is more variable and noisy [19].

The variability in performance is also influenced by the population studied. In high-risk groups, such as those with a history of self-injury, AI models can achieve higher predictive accuracy. In contrast, in more general populations, where risk factors are more diffuse, the accuracy tends to be lower [20]. Additionally, certain ML models, such as Random Forest and neural networks, have been shown to perform better when trained on longitudinal datasets that capture temporal patterns in behavior [12].

Additionally, integrating AI with traditional clinical data in the United States has improved risk prediction accuracy to a further extent [21], offering a substantial enhancement over conventional approaches.

The potential of AI in suicide prevention extends beyond prediction. AI-driven interventions, such as chatbots and virtual therapists, are being developed to provide immediate support and resources to individuals in crisis [22,23]. These tools can offer a bridge to professional care, especially in regions with limited access to mental health services. For example, in rural areas of India, AI-powered chatbots have been implemented to provide mental health support [24], showing promising results in reducing distress and preventing escalation.

The primary objective of this review is to critically examine the role of AI and ML technologies in improving the accuracy and reliability of suicide prediction models, specifically focusing on their ability to incorporate complex risk factors such as psychiatric conditions, socio-economic influences, dietary patterns, and environmental contaminants. In particular, we explore how AI and ML models can integrate these factors to enhance the precision of suicide risk assessments and provide data-driven strategies for effective prevention interventions. Additionally, this review highlights AI-driven interventions, such as virtual therapists and intelligent monitoring systems, that offer immediate support to at-risk individuals, particularly in regions with limited access to mental health services. We hypothesize that incorporating diverse data sources, including dietary and environmental information, will significantly enhance the predictive accuracy of AI models. This review also discusses the ethical and privacy concerns related to AI applications in suicide prevention, proposing frameworks for responsible deployment.

## 2 Methodology

### 2.1 Search strategy and selection criteria

In this review, the methodology involved a structured approach to identify and gather relevant literature on the role of AI and ML in suicide prediction and prevention, alongside studies exploring the influence of dietary patterns and environmental contaminants on suicidal behaviors. The primary databases searched included PubMed, Web of Science, and IEEE Xplore, ensuring a comprehensive interdisciplinary overview of the topic.

The search strategy was based on specific keywords and phrases designed to capture relevant studies across various fields. These keywords included combinations of the following terms: ["artificial intelligence," "machine learning," "suicide prediction," "mental health AI applications," "dietary influences," "environmental contaminants and suicide"]. Each term was used in conjunction with Boolean operators to maximize the breadth and depth of the search.

The search primarily focused on studies published between 2010 and 2024, reflecting the rapid advancements in AI/ML technologies during this period. However, foundational studies published before 2010 were selectively included when they provided significant insights into key epidemiological or methodological aspects relevant to suicide research.

By detailing these methods, we aim to provide a clear framework that supports the replicability of our review process and aids in understanding the scope and limitations of the gathered data. While this review is not systematic, it adheres to robust methodological standards to ensure that the conclusions drawn are well-supported and meaningful for future research and practical applications in pediatric healthcare.

## 3 Discussion

### 3.1 Influence of food and drinks on suicidal tendencies

The relationship between dietary habits and mental health has gained considerable attention, particularly concerning suicidal behaviors. Various studies highlight how certain foods and drinks can influence the risk of suicide, either increasing or decreasing this risk [[25], [26], [27]].

Caffeine consumption, particularly in moderate amounts, has been linked to a reduced suicide risk in women [28], though no significant change is observed in men when adjusted for other factors like age and depression [28]. In contrast, the combined use of alcohol and energy drinks significantly raises the likelihood of suicidal ideation and attempts [10]. High soft drink consumption, especially three or more times daily, is associated with increased suicidality in both the U.S [29]. and China [27], highlighting a global pattern. This suggests that certain chemical components in soft drinks may have adverse neurobiological effects, potentially exacerbating mood disorders [30].

In general, food and drinks, depending on their chemical structure and compositions, might have a particular chemical mechanism that influences the body in a way that is similar to synthetic drugs. For instance, high sugar intake from sweetened beverages may disrupt glucose metabolism and lead to mood destabilization, increasing suicidal risk [31].

Dietary patterns also play a crucial role. A diet rich in vegetables, fruits, and fish, known as the prudent dietary pattern, is associated with a decreased risk of suicide [32]. Conversely, high intake of sweet foods and fast food is linked to an increased risk of suicide attempts [27], particularly among adolescents. Specific foods like goat meat have shown a negative association with suicide attempts [25], while pork consumption correlates positively [25]. The mechanisms behind these associations are not fully understood, but it is speculated that goat meat, which is leaner and contains fewer inflammatory fats, may promote mental health [33], whereas pork may increase systemic inflammation, which has been linked to depressive symptoms and suicidal ideation [34,35].

Nutritional components such as polyunsaturated fatty acids (PUFAs) and vitamins also impact suicidal tendencies. Higher levels of arachidonic acid (AA), a type of PUFA, serve as a protective factor against suicide attempts [36], especially in individuals with a history of such attempts. This may be due to AA's role in modulating inflammation and supporting neural health. Vitamin D deficiency has been linked to increased risk, with supplementation potentially mitigating this risk by enhancing serotonin synthesis [37]. Other factors, such as smoking [38] and dietary fiber intake [39], further illustrate the complex interplay between nutrition and suicidal behavior. For example, smoking has been linked to altered neurotransmitter levels and oxidative stress, both of which may increase vulnerability to suicidal thoughts [40], while high fiber intake might contribute to gut health and, consequently, improved mental well-being [41]. Among others, these relationships are highlighted in Table 1, providing a concise overview of various food and drink factors and their impact on suicidal tendencies.

**Table 1:** Influence of food and drink factors on suicidal tendencies.

Factor	Impact on Suicidal Tendencies	Reference
Caffeine Consumption	Regular and moderate caffeine intake likely reduces suicide risk in women, but no change was observed in men after statistical adjustment for age, depression, and sleep problems.	[28]
Alcohol and Energy Drink Use	Combination use and heavy alcohol use increase the likelihood of suicidal ideation or attempts.	[10]
Soft Drink Consumption (U.S.)	Consumption of ≥3 times/day is significantly associated with an increased risk of suicidality, including suicidal ideation, suicide plan, suicide attempt, and suicide attempt with medical treatment, regardless of other factors.	[29]
Soft Drink Consumption (China)	≥3 times/day increases the risk of suicidal ideation, plan, and attempt by 80 % and 3.5-fold, respectively. Nonconsumption of soft drinks was also associated with about 32 % elevated risk for suicidal plans and suicidal attempts.	[27]
Sweet food intake	high sweet food intake increases risk.	[27]
Fast Food Consumption	Positively associated with increased risk of suicide attempts among adolescents.	[100]
Prudent Dietary Pattern	Associated with a decreased risk of suicide (HR 0.46, 95 % CI 0.28–0.75); includes high intake of vegetables, fruits, potatoes, soy products, mushrooms, seaweed, and fish.	[32]
Goat Meat Consumption	Negatively associated with a history of suicide attempts (OR = 0.39).	[25]
Pork Consumption	Positively associated with a history of suicide attempts (OR = 2.35).	[25]
Traditional Japanese Diet Score (TJDS)	No significant association with suicide rates before the year 2000. A significant negative association after 2000, indicating a protective factor against suicide.	[101]
Polyunsaturated Fatty Acids (PUFAs)	Higher arachidonic acid (AA) is a protective factor against suicide attempts, particularly in those with a prior attempt and major depressive disorder. No significant impact from eicosapentaenoic acid (EPA) or docosahexaenoic acid (DHA).	[36]
	Higher intake of n-3 relative to n-6 PUFAs may reduce depression and suicide risk, particularly in women.	[102]
Cholesterol	Total LDL- and HDL-cholesterol levels did not predict subsequent suicide events.	[36]
Vitamin D	Vitamin D deficiency may increase the risk of depression and suicide; supplementation may reduce this risk by enhancing serotonin synthesis and modulating proinflammatory cytokines.	[37]
Antioxidant Vitamins and Carotenoids	Low levels of antioxidant vitamins (e.g., vitamin C) and carotenoids (e.g., α-carotene, β-cryptoxanthin, lutein/zeaxanthin, lycopene) are associated with a higher prevalence of suicide attempts.	[103]
Folic Acid (Vitamin B9)	Consumption of folic acid, as indicated by prescription fills, is associated with a decreased risk of suicide attempts and intentional self-harm (HR: 0.56).	[104]
Fruit and Vegetable (F&V) Intake	F&V intake is associated with a lower risk of suicidal ideation. Non-consumers had an increased risk for suicidal ideation compared to consumers.	[26]
Unwashed Raw Vegetables Consumption	Positively associated with a history of suicide attempts (OR = 3.23).	[25]
Sugar-Sweetened Beverages (SSBs)	Consumption of ≥1 SSB/day is associated with a higher risk of suicidal ideation (OR: 2.20).	[105]
Fiber Intake	Lower intake of dietary fiber is associated with a history of suicide attempts.	[39]
Former smokers	31 % increased risk of death by suicide (RR = 1.31), 35 % increased risk of suicidal ideation (RR = 1.35), and 27 % increased risk of suicide attempts (RR = 1.27)	[38]
Current smokers	141 % increased risk of death by suicide (RR = 2.41), 84 % increased risk of suicidal ideation (RR = 1.84), and 71 % increased risk of suicide attempts (RR = 1.71)	[38]
Smoking/Gender differences	Current smoker women: 151 % increased risk of death by suicide (RR = 2.51); Current smoker men: 106 % increased risk of death by suicide (RR = 2.06)	[38]
Overall smoking exposure	74 % increased risk of suicidal behavior, including death by suicide, ideation, planning, or attempts (RR = 1.74)	[38]

### 3.2 Impact of water/food contaminants on suicidal tendencies

The relationship between contaminants in water and food and their impact on suicidal tendencies has garnered increasing attention. Research indicates that various contaminants can either mitigate or exacerbate suicide risk. For example, higher levels of lithium in public drinking water are associated with lower suicide rates in men [11], suggesting a potential protective effect. Similarly, arsenic concentrations in drinking water have been negatively associated with suicide rates in both men and women, despite its known health risks [42]. In contrast, mercury exposure across generations has been linked to an increased risk of suicide attempts among children and youth [43]. Fluoride in drinking water shows a negative correlation with suicide rates, indicating that higher fluoridation levels might reduce suicide risk [44]. Chromium does not show a significant association with suicide rates in the general population but does have a protective effect in certain demographics [45]. Additionally, exposure to organophosphorus pesticides, specifically dimethylthiophosphate, has a positive association with suicidal ideation, especially in young and older men [46]. These findings highlight the complex and varied effects of different contaminants on mental health and suicidal behavior as shown in Table 2.

**Table 2:** Impact of water contaminants and substances on suicidal tendencies.

Contaminant	Impact on Suicidal Tendencies	Reference
Lithium	Higher levels of lithium in public drinking water are associated with lower suicide rates in men. After adjusting for gender distribution, the inverse correlation remained statistically significant in men but not in women.	[11]
Arsenic	Arsenic concentrations in drinking water (averaging 0.969 μg/L, CI: 0.543–1.396 μg/L) were negatively associated with suicide rates in both men and women. This suggests a potential protective effect of arsenic on suicide, despite increased mortality from natural causes.	[42]
Fluoride	The study found that the percentage of people drinking fluoridated water was negatively correlated with suicide rates (partial correlation = −0.24, p = 0.05), indicating that higher fluoridation levels were associated with lower suicide rates.	[44]
Mercury	Mercury exposure across three generations is linked to increased risk of suicide attempts among children (5–11 years old) and youth (12–17 years old).	[43]
Chromium	No statistically significant association with suicide rates in the general population (p = 0.35, r = −0.12). However, a significant inverse relationship was found between chromium concentration and suicide deaths in whites (p = 0.009, r = −0.32), suggesting a potential protective effect in this demographic.	[45]
Exposure to Organophosphorus Pesticides (OPPs)	Exposure to dimethylthiophosphate (DMTP) among OPPs demonstrated a statistically significant positive association with suicidal ideation (SI) [OR: 1.18; 95 % CI: 1.02–1.37]. Stratified analyses showed that this influence was particularly pronounced in young and older men.	[46]

### 3.3 Limitations of conventional suicide risk assessment

Conventional methods used to assess suicide risk, such as clinical interviews, patient self-reports, and standardized tools like the Columbia-Suicide Severity Rating Scale (C-SSRS) and the Patient Health Questionnaire-9 (PHQ-9), have notable limitations. These methods can be subjective [47,48], as they rely on patients' willingness and ability to communicate their mental state. This reliance can sometimes result in inconsistent care due to the variability in clinical judgment.

One significant drawback is that these assessments only provide a brief glimpse into a patient's mental state during clinical visits [49], often failing to capture the ever-changing nature of suicidal thoughts and actions. It is important to note that not all individuals who die by suicide show obvious warning signs during their last clinical encounter [50], which emphasizes the limitations of intermittent evaluations.

In addition, conventional approaches fail to consider the intricate interaction of various factors that contribute to the risk of suicide. Conventional assessment tools like the PHQ-9 do not fully encompass all the factors that contribute to risk, including genetic predispositions, real-time social stressors, and subtle behavioral changes [51,52]. This limitation is especially evident in diverse populations, where cultural and contextual differences impact the manifestation and understanding of suicidal symptoms [53]. Table 3 provides an overview of conventional suicide risk assessment methods and their limitations.

**Table 3:** Limitations of conventional suicide risk assessment methods.

Method	Limitations	References
Clinical Interviews	Subjective; dependent on clinician's skill and experience; variability in interpretation	[106]
Patient Self-Reports	May not capture true risk due to underreporting or dishonesty; influenced by patient's current mood	[14]
Standardized Questionnaires	Limited scope; focus on psychological symptoms; may not account for social or biological factors	[107]
Columbia-Suicide Severity Rating Scale (C-SSRS)	Episodic; only captures risk at the time of assessment; requires honest disclosure from the patient	[108,109]
Beck Scale for Suicide Ideation (BSS)	Not predictive; primarily assesses current ideation; lacks continuous monitoring capabilities	[110]
PHQ-9 (Patient Health Questionnaire-9)	General depression screening; not specific to suicide risk; may miss non-depressive suicidality indicators	[51,52]
Sad Persons Scale	Simplistic scoring; limited predictive validity; does not consider dynamic risk factors	[111]
Risk Scales and Checklists	Often have low predictive value; static assessments that do not capture changing risk levels	[112]
Hamilton Depression Rating Scale (HDRS)	Primarily measures depression severity; not specific to suicidal thoughts or behaviors	[113]
Geriatric Depression Scale (GDS)	Designed for older adults; may not be applicable to younger populations; focuses on depression rather than suicidality	[114]
Clinical Global Impression (CGI)	Subjective measure; lacks specificity for suicide risk assessment, do not refer to specific symptoms other than suicidal thoughts	[115]
Youth Risk Behavior Surveillance System (YRBSS)	Self-reported data from adolescents; subject to underreporting and recall bias	[116]
Patient Health Questionnaire for Adolescents (PHQ-A)	Adapted from PHQ-9 for adolescents; still primarily focused on depression	[117]
Risk of Suicide Questionnaire (RSQ)	May have poor sensitivity to the components of attachment style most important in eliciting emotional responses.	[118]
Behavioral Health Screen (BHS)	It may be time-consuming and require significant resources	[119]
Mini International Neuropsychiatric Interview (MINI)	Structured diagnostic interview; time-consuming and requires trained interviewers	[120]

The need for data-driven, predictive approaches is clear. Advances in AI and machine learning ML offer promising solutions. AI technologies can identify patterns and risk factors that conventional methods may miss, providing advanced tools for suicide prediction and supporting the development of tailored suicide prevention interventions [54]. AI models have demonstrated high accuracy in predicting suicide attempts by integrating multifaceted data streams, surpassing the predictive power of conventional tools [54].

### 3.4 Transformative artificial intelligence and machine learning technologies in mental health

The applications of AI and ML are revolutionizing healthcare, particularly in mental health, through innovative advancements in diagnosis, treatment, and patient management. AI and ML technologies enable computers to learn from vast datasets, identify patterns, and make informed decisions with minimal human intervention [55,56]. In mental health, these technologies are transformative.

In diagnosis, AI algorithms can analyze EHRs that might consider some questions related to the type of food and drinks and elements that have the possible composition and a significant role in suicide behavior to identify patterns indicative of mental health conditions [17]. For example, AI systems like IBM Watson Health have been used to detect signs of depression and anxiety from patient records with high accuracy [57]. Social media analysis is another area where AI excels; systems can monitor posts for language and sentiment changes that may indicate suicidal ideation, as seen with AI tools used by platforms like Facebook, such as a study leveraged social media images to predict suicide risk using interpretable models [58]. By analyzing 177,220 images that 841 Facebook users uploaded, the model achieved high prediction performance (AUC = 0.720), demonstrating the potential of using visual data for suicide risk assessment [58]. Studies using platforms like Reddit and Twitter have employed models such as Long Short-Term Memory - Convolutional Neural Networks (LSTM-CNN), achieving high accuracy in detecting suicidal posts by analyzing language patterns and sentiment changes [59]. Models incorporating historical context, such as the STATENet model, use time-aware transformers to analyze the progression of emotional states over time. This approach has improved the accuracy of detecting suicidal intent by considering the user's activity history on social media [60].

Predictive analytics enhance care by forecasting mental health crises. For instance, researchers at Vanderbilt University developed an ML model that predicts the likelihood of suicide attempts by analyzing EHR data. This model predicted suicide risk with an accuracy rate of 84–92 % within 1 week and 80–86 % within 2 years [61]. Such models enable proactive interventions, potentially preventing severe outcomes by identifying at-risk individuals before a crisis occurs.

Another study by Guille et al., has discussed a web-based CBT program delivered before the start of the internship year significantly reduced suicidal ideation among medical interns, showing a 12 % incidence in the intervention group versus 21.2 % in the control group [62]. This demonstrates the potential of preventative measures for high-risk groups.

Researchers developed the "Suicide Artificial Intelligence Prediction Heuristic (SAIPH)" algorithm to predict suicidal thoughts by analyzing Twitter data related to stress, loneliness, and depression [63]. Training on over 512,000 tweets from suicidal individuals and 3.5 million control tweets, the model achieved an AUC of 0.88. SAIPH can predict suicidal ideation with a sevenfold increased risk within 10 days of peak occurrences [63]. Validation showed strong correlations with regional suicide rates, especially among younger individuals, demonstrating its potential for suicide risk monitoring and screening [63]. These advancements in mental health care driven by AI and ML have the potential to enhance patient outcomes and improve the efficiency of healthcare systems.

### 3.5 Machine learning and deep learning for suicide prediction

The applications of ML and deep learning (DL) are revolutionizing suicide prediction by enhancing accuracy and early detection [64]. The ML algorithms like K-Nearest Neighbor (KNN) and Random Forest analyze complex datasets to enhance suicide prediction and guide subsequent prevention efforts by identifying at-risk individuals more effectively [65]. Such methods should obviously have an algorithm program that focuses on the results of EHRs in a way to encounter the possible suicide danger. A study utilized Random Forest algorithms to analyze the electronic health records of 5167 adult patients who had self-injured. The model accurately predicted future suicide attempts in 3250 patients with a precision of 79 % and recall of 95 %, significantly improving the ability to tailor interventions to individuals most at risk [66]. This personalized approach ensures that mental health resources are directed where they are needed most.

Another study was conducted by Haines-Delmont et al., which utilized KNN algorithms to analyze smartphone data collected from 66 inpatients with acute mental health conditions [56]. The data included sleep behavior, mood, step frequency, and engagement patterns with mobile devices. The KNN model, with k = 2 and uniform weighting, predicted suicide risk with 68 % mean accuracy [56]. This approach demonstrated the potential for continuous and accurate suicide risk assessment, significantly improving early intervention and resource allocation to individuals most at risk.

Deep learning models, such as Convolutional Neural Networks (CNNs) and Recurrent Neural Networks (RNNs), further enhance predictive accuracy. CNNs analyze visual data from social media to detect indicators of suicidal ideation [67], while RNNs process sequential data like speech patterns and online behavior to identify risks over time [68].

Neural network models using EHR for predicting suicide attempts in adolescents have shown high accuracy. A study using data from California-resident adolescents demonstrated that a depth-4 neural network model achieved a sensitivity of 0.703, specificity of 0.980, and AUC of 0.958 [69].

Combining Ecological Momentary Assessments (EMA) with EHR data in deep sequential models improved recall in predicting suicidal ideation from 48.13 % to 67.78 %. This approach used recurrent neural networks (RNNs) to align and analyze data sequences [70].

Various ML and DL algorithms have been employed to enhance the accuracy and early detection of suicidal behaviors. These algorithms analyze complex datasets to identify patterns associated with suicide risk. Table 4 provides an overview of these algorithms and their specific applications in suicide prediction.

**Table 4:** Machine learning and deep learning algorithms for suicide prediction.

Algorithm Type	Algorithm Name	Application	Example in Suicide Prediction	Reference
Machine Learning	K-Nearest Neighbor (KNN)	Similarity-based classification	Analyzed smartphone data from inpatients to predict suicide risk with 68 % accuracy	[56]
Machine Learning	Random Forest	Ensemble decision trees	Analyzed electronic health records to predict future suicide attempts with 79 % precision and 95 % recall	[66]
Machine Learning	Smooth Support Vector Machine (SVM)	Medical record classification	Used Smooth SVM to predict suicide-related behaviors with 63 % accuracy using psychiatric patient records	[121]
Machine Learning	Support Vector Machine (SVM)	Psychological stress analysis	Utilized SVM to analyze psychological stress factors and predict suicidal thoughts	[122]
Machine Learning	Support Vector Machine (SVM)	Mobile data analysis	Tested SVM on smartphone data to predict suicide risk among inpatients	[56]
Tree-based	Classification Tree	Decision tree analysis	Identified adolescent suicide attempters with 69.8 % sensitivity, 85.7 % specificity	[123]
Tree-based	Random Forest	Ensemble decision trees	Improved suicide risk prediction using temporal variables with AUC = 0.824, 0.339 sensitivity at 95 % specificity	[124]
Tree-based	Classification Tree	Decision tree analysis	Predicted suicidal ideation in older adults with 81 % AUC, 25 % ideation in high distress, 50 % in high distress and low function	[125]
Machine Learning	Naive Bayes	Probabilistic classification	Improved suicide risk prediction with an AUC of 0.754 compared to other models	[124]
Machine Learning	Logistic Regression	Binary outcome regression	Used to classify suicide attempters among patients with schizophrenia with 67 % accuracy and an AUC of 0.71	[126]
Machine Learning	Gradient Boosting	Ensemble of weak prediction models	Predicted suicide ideation and attempts in a general population with an AUC of 0.80	[127]
Deep Learning	Convolutional Neural Networks (CNNs)	Visual data analysis	Detects suicidal indicators from social media images	[58]
Deep Learning	Recurrent Neural Networks (RNNs)	Sequential data analysis	Monitors speech patterns and behavior over time	[68]
Deep Learning	Long Short-Term Memory (LSTM)	Long-term dependency analysis	Analyzes time-series data for suicidal thoughts	[59]
Neural Networks	Recurrent Neural Networks (RNNs)	Sequential data modeling	Improved prediction recall of suicidal ideation from 48.13 % to 67.78 % by integrating EMA records with EHR data	[70]
Deep Learning	CNN Autoencoder	Brain structural imaging analysis	Employed to predict suicidal ideation based on brain imaging data with an accuracy of 85 %	[128]
Neural Networks	Multilayer Perceptron (MLP)	Feedforward classification	Combines features to predict suicide attempts	[129]
Neural Networks	Deep Belief Networks (DBNs)	Sentiment analysis on social media	Used to predict suicidal ideation in students by analyzing sentiment text from social media posts with high accuracy	[130]
Ensemble Methods	Bagging	Model aggregation	Combines multiple machine learning models to predict suicide attempts with high accuracy among patients with depression	[131]
Deep Learning	TransformerRNN	Sequence modeling	Analyzes long-term text dependencies to identify suicide notes with high accuracy on social media	[132]

### 3.6 Natural Language Processing in suicide detection

**Fig. 1:**
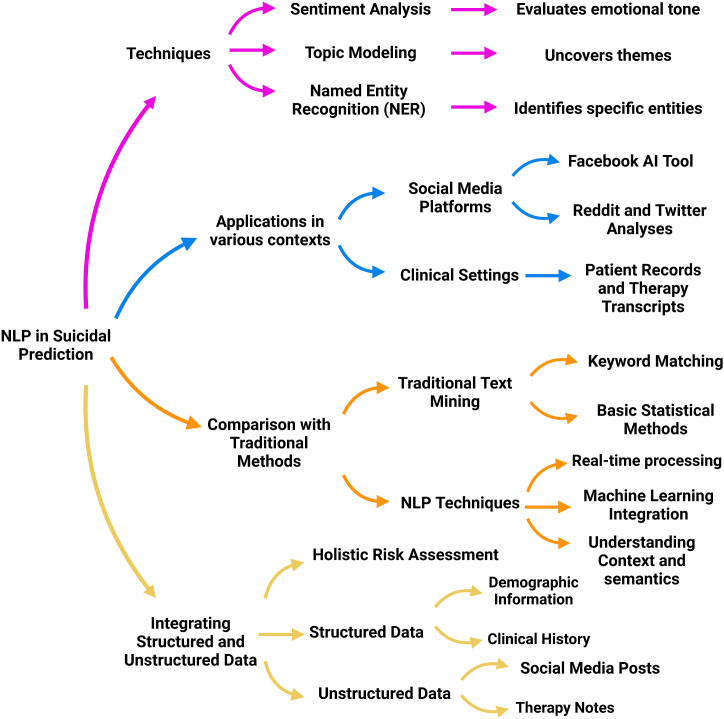
Mind map illustrating NLP techniques, applications, comparison with traditional methods, and integration of structured and unstructured data for suicide detection.

Natural Language Processing (NLP) has become a critical tool in suicide detection, utilizing advanced techniques to analyze textual data from diverse sources [71]. Techniques such as sentiment analysis [72], topic modeling [73], and named entity recognition (NER) [74] have shown significant potential in identifying language patterns associated with suicidal ideation. These key techniques and their applications are shown in Fig. 1.

Sentiment analysis evaluates the emotional tone of text data [72], identifying distress or hopelessness, while topic modeling uncovers themes like isolation or substance abuse from forums or therapy transcripts [75]. NER enhances the granularity and accuracy of risk assessments by identifying specific entities within the text and providing detailed linguistic insights [76].

NLP applications in suicide detection span various contexts, including social media platforms and clinical settings. Platforms like Facebook and Twitter employ NLP algorithms to monitor user posts for signs of distress, enabling timely intervention and suicide prevention efforts [77,78]. For instance, Facebook's AI tool scans posts and comments to identify at-risk users and alert crisis intervention teams [79]. Studies using Reddit and Twitter analyses have also achieved significant success in real-time monitoring and response [80]. In clinical settings, NLP analyzes patient records and therapy transcripts, processing unstructured data from EHRs to identify patients at risk based on historical and real-time data [81]. This application has shown promise in improving early detection and intervention strategies.

Traditional text mining techniques, relying on keyword matching and basic statistical methods, are often limited in detecting nuanced expressions of suicidal intent [82]. In contrast, NLP offers a sophisticated approach to understanding the context and semantics of language [83]. NLP models, particularly those incorporating machine learning, can learn from vast amounts of data and adapt to new linguistic patterns, providing a dynamic and accurate assessment [84]. NLP's ability to process and analyze large volumes of unstructured text in real-time enhances its utility over traditional methods [85], making it a superior tool for continuous monitoring and intervention.

Integrating structured and unstructured data is crucial for comprehensive suicide risk assessment. Structured data, such as demographic information and clinical history, provides foundational context [86]. In contrast, unstructured data, like social media posts and therapy notes, offers real-time insights into a patient's mental state [87]. NLP models synthesize these diverse data sources to create a holistic view of an individual's risk profile [88]. Combining EHR data with social media analysis reveals patterns and correlations that may not be evident from structured data alone, enhancing the predictive accuracy of NLP models and enabling more timely and personalized interventions [89].

### 3.7 Ethical and privacy considerations in AI-based suicide prevention

Ensuring the ethical and privacy aspects of integrating AI in suicide prevention is crucial to protecting the rights and well-being of individuals. It is of utmost importance to prioritize the protection of data privacy and security. AI systems must comply with strict data protection regulations, such as GDPR or HIPAA, to safeguard sensitive health information from breaches and misuse [90]. Ensuring confidentiality requires the use of encryption, anonymization, and secure data storage [90]. Addressing ethical dilemmas in AI applications involves navigating issues such as informed consent, potential biases, and the implications of AI-driven interventions [91,92]. AI models must be developed with a clear ethical framework, ensuring that individuals are fully aware of how their data will be used and the potential consequences of AI predictions [93,94]. Mitigating biases in AI algorithms is crucial, as biased models can lead to unfair treatment and exacerbate existing disparities in mental health care. Transparent and explainable AI is of utmost importance. AI systems utilized in suicide prevention should offer transparent and comprehensible justifications for their predictions and interventions [90]. This level of transparency helps to build trust between users and clinicians, establishing AI as a dependable and helpful tool in the field of mental health care.

The implementation of AI in suicide prevention within resource-limited settings presents several challenges. Firstly, infrastructure limitations, such as unreliable internet access and limited computing power, hinder the deployment of advanced AI technologies [95]. Developing mobile-based AI platforms capable of operating offline can help overcome these barriers in remote areas.

Also, the quality of available data is often compromised by inconsistent reporting and stigma surrounding suicide in these regions [96]. AI models need to be adaptable to such data constraints by using techniques like transfer learning and federated learning, ensuring accurate predictions without requiring large, high-quality datasets.

Furthermore, cost remains a significant hurdle. The development, deployment, and maintenance of AI systems can be expensive, particularly in low-resource settings [97]. Therefore, open-source, low-cost AI solutions are essential to integrate AI into existing healthcare systems without overwhelming financial resources.

Cultural sensitivity is also crucial. Mental health behaviors and suicide risk factors vary significantly across regions, and AI models must be rigorously validated to account for these cultural differences [98,99]. Without this, there is a risk of biased or inaccurate predictions that may harm rather than help the target populations.

It is crucial to integrate ethical and privacy considerations to fully utilize the power of AI in suicide prevention. This will guarantee that technological progress leads to secure, equitable, and efficient mental health interventions.

## 4 Conclusion

This review highlights the transformative role of AI and ML in suicide prediction and prevention, surpassing traditional methods that rely on subjective assessments. AI models demonstrate superior predictive capabilities by integrating diverse datasets from electronic health records, social media, dietary patterns, and environmental exposures. Our detailed examination of dietary and environmental factors revealed critical interactions, such as the protective effects of moderate caffeine consumption and lithium in water, contrasted with the risks associated with pork consumption and mercury exposure. These findings emphasize the importance of geographically and demographically tailored public health interventions. In terms of methodology, our review has analyzed the integration of AI and ML models in predicting suicide risks, focusing on their capacity to incorporate multifactorial data sources, including psychiatric, social, dietary, and environmental factors. This comprehensive approach supports the development of more accurate and practical models. However, significant limitations remain. Ethical concerns, particularly regarding data privacy, algorithmic bias, and the transparency of AI systems, present substantial limitations. Moreover, the availability of high-quality, diverse datasets across different populations remains a key challenge for enhancing predictive accuracy. Future research should prioritize the optimization of AI models by integrating more granular, multi-dimensional datasets that include precise dietary and environmental variables. Additionally, efforts must focus on mitigating algorithmic biases, enhancing model generalizability, and improving predictive precision to facilitate the development of personalized and effective suicide prevention interventions.

## CRediT authorship contribution statement

**Mayyas Al-Remawi:** Writing – original draft, Conceptualization. **Ahmed S.A. Ali Agha:** Writing – original draft, Conceptualization. **Faisal Al-Akayleh:** Writing – review & editing. **Faisal Aburub:** Writing – review & editing. **Rami A. Abdel-Rahem:** Writing – review & editing.

## Ethics approval and consent to participate

Not applicable. This article contains no studies performed by authors with human participants or animals. It is a comprehensive review, synthesizing insights from previously published articles.

## Data availability statement

No data was used for the research described in the article.

## Declaration

We confirm that this manuscript is not under consideration elsewhere and that all authors have consented to its submission.

## Declaration of competing interest

The authors declare that they have no known competing financial interests or personal relationships that could have appeared to influence the work reported in this paper.
